# Auditory Brainstem Response Recorded with the NeuroAudio System in Children Under 3 Years of Age

**DOI:** 10.3390/life16071044

**Published:** 2026-06-23

**Authors:** Milaine Dominici Sanfins, Diego Lourenço dos Santos Silva, Rhayane Vitória Lopes, Emilia Czaplicka, Piotr Henryk Skarzynski

**Affiliations:** 1 Department of Speech-Hearing-Language, Universidade Federal de São Paulo, São Paulo 04044-02, Brazil; 2 Department of Teleaudiology and Screening, World Hearing Center, Institute of Physiology and Pathology of Hearing, 05-830 Kajetany, Poland; 3 Department of Otolaryngology, Institute of Sensory Organs, 05-830 Warsaw, Poland

**Keywords:** auditory brainstem response, reference values, infant, hearing tests, interaural asymmetry

## Abstract

Background: The click-evoked Auditory Brainstem Response (ABR) is the gold standard electrophysiological tool for assessing auditory pathway integrity in infants and young children. As normative data are inherently equipment-specific, the absence of pediatric reference values for the NeuroAudio system (Neurosoft, Ivanovo, Russia) represents a significant gap in clinical practice, given that existing normative datasets for this system are restricted to adult populations. Objective: To establish normative data for click ABR recorded with the NeuroAudio system in children under three years of age, stratified by age group according to auditory maturation patterns. Methods: A prospective, cross-sectional study was conducted at the Electrophysiology Laboratory of the Department of Speech Therapy, Paulista School of Medicine, Federal University of São Paulo (UNIFESP/EPM), under the approval of the Research Ethics Committee (protocol 7.939.564). A total of 203 children (121 males, 82 females; age range: 2 weeks to 36 months) with confirmed normal peripheral auditory function were included. Click stimuli (0.1 ms, rarefaction polarity) were delivered monaurally via ER-3A insert earphones at 80 dB nHL and a repetition rate of 19.3/s. Two average runs of 2000 artifact-free sweeps were recorded per ear. Absolute latencies of waves I, III, and V, interpeak intervals I–III, III–V, and I–V, and amplitudes of waves I and V were analyzed. Results: Statistical modeling supported the consolidation of 12 initial age bins into three clinically and statistically validated categories: 0–3, 4–12, and 13–36 months. Wave I latency remained stable across age groups, whereas waves III and V and all interpeak intervals showed progressive shortening with increasing age. Wave V amplitude increased progressively with age, while wave I amplitude remained unchanged. Females presented shorter latencies than males for waves III and V and for all interpeak intervals. The right ear exhibited a shorter III–V interpeak interval than the left ear, with a significant ear × age interaction indicating that this asymmetry is modulated during early maturation. Age, sex, and ear-stratified normative values (two SD and three SD reference limits) are reported. Conclusion: This study provides the first pediatric normative dataset for click-evoked ABR acquired with the NeuroAudio system in children under three years of age. The proposed three age stratifications, together with sex- and ear-specific reference values for the III–V interpeak interval, offer a clinically actionable framework for the accurate interpretation of pediatric ABR recordings and for the early identification of auditory pathway abnormalities.

## 1. Introduction

The evaluation of Auditory Brainstem Response (ABR) with click-type stimuli is an extremely important tool in pediatric diagnosis, especially in young children who are not always able to cooperate with behavioral tests.

Pediatric audiological diagnosis aims to identify hearing loss early and should be based on the principle of the cross-check, that is, the evaluations (behavioral, physiological, and electrophysiological) need to be consistent with the results obtained. It should be noted that no audiological evaluation should be concluded with the completion of just one procedure [1,2,3]. Aiming to encompass the principle of the cross-check, different international and national entities have established guidelines and recommendations on screening, diagnosis, and audiological monitoring methods for pediatric audiological assessment [4,5]. Thus, it is suggested that pediatric audiologists use protocols based on scientific evidence and in accordance with the criteria recommended by international and national bodies, since the protocols must consider the socioeconomic and cultural context of the locality where they will be applied [4].

It is important that the requesting physicians and evaluators analyze the strengths and limitations of each audiological procedure and consider them in the development of a battery of diagnostic tests specific to the patient. The methodology considered the gold standard in audiological investigation up to six months of age is the Auditory Brainstem Response (ABR) for both the analysis of the integrity of the auditory pathway (click ABR) and the assessment of auditory thresholds [specific frequency ABR (SF-ABR)] [6,7]. The electrophysiological evaluation can be performed with the child in natural sleep, with sedation reserved for those individuals who could not undergo the examination under satisfactory conditions [8].

Regarding the evaluation of the click ABR, it is relevant to mention that the click stimulus, due to its acoustic particularities, is not the best option for investigating auditory thresholds. Since the year 2000, Stapells has reported that the research of electrophysiological thresholds through the click ABR would only be successful if the patient presented a hearing loss with a horizontal configuration, as the click-type stimulus stimulates a large area of the cochlea and, thus, it cannot be precisely determined which frequency represents the threshold of the click ABR [7]. However, up to the present moment, the click ABR is the best procedure for analyzing the integrity of the auditory pathway up to the brainstem region and should be performed before any other procedure aimed at assessing the audibility of an infant, baby, or child [9,10].

The clinical interpretation of the click ABR relies on three robust components, waves I, III, and V, whose anatomical generators are distributed along the ascending auditory pathway. Wave I originates in the distal portion of the auditory (eighth cranial) nerve; wave III is generated in the caudal brainstem, at the level of the cochlear nucleus and superior olivary complex; and wave V reflects activity in the rostral brainstem, arising from the lateral lemniscus and its termination in the region of the inferior colliculus [11,12,13]. Because these structures undergo myelination and synaptic refinement at different rates during the first years of life, the absolute latencies of waves I, III, and V and the corresponding interpeak intervals (I–III, III–V, and I–V) constitute sensitive indices of the maturational state of successive segments of the auditory pathway. For this reason, these three components, rather than the more variable waves II and IV, are the ones consistently monitored in pediatric ABR and constitute the focus of the present normative investigation.

As a result of the maturational process that seems to stabilize in the brainstem around 24–36 months of age, it is essential to have well-established normative criteria for different age groups in the evaluation of click ABR [6,9,10]. An interesting study that is one of the major references for the maturational process based on click ABR was conducted by Gorga and his collaborators. The Boys Town Auditory Brainstem Response presented the normal values for the responses of the click ABR assessment in individuals from 33 to 34 weeks of life to 36 months of age, allowing this assessment model to be widely employed around the world [14,15].

However, with the emergence of new auditory evoked potential instruments on the market and around the world, it has become necessary to conduct new studies in order to provide normative data for the pediatric population. Thus, the objective of the present study is to present normative data for the click ABR in individuals under three years of age with hearing within normal limits using the NeuroAudio system.

## 2. Material and Methods

### 2.1. Ethics Statement

The present study, with a prospective and cross-sectional design, received approval from the Research Ethics Committee (REC) of the Federal University of São Paulo (UNIFESP), as recorded under protocol number 7.939.564. All participants’parent or legak guardians provided written Informed Consent Forms (ICFs). Data collection was conducted at the facilities of the Electrophysiology Laboratory of the Department of Speech Therapy at the Paulista School of Medicine, Federal University of São Paulo (UNIFESP/EPM).

### 2.2. Participants

The study was composed of 203 individuals initially divided into 12 age groups representing consecutive three-month intervals spanning from birth to 36 months (0–3, 3–6, 6–9, 9–12, 12–15, 15–18, 18–21, 21–24, 24–27, 27–30, 30–33, and 33–36 months). The participating individuals met all eligibility criteria: (1) age below 36 months (3 years); (2) normal bilateral otoscopy; (3) transient otoacoustic emissions within normal limits in both ears [16,17,18]; (4) type A tympanograms in both ears [17] performed with a 1000 Hz probe tone; (5) ipsilateral acoustic reflexes present in both ears with a 1000 Hz probe tone in response to pure tones of 500, 1000, 2000, and 4000 Hz for babies up to 6 months old [18,19,20]; (6) ipsilateral acoustic reflexes present in both ears with a 226 Hz probe tone in response to pure tones of 500, 1000, 2000, and 4000 Hz for babies over 6 months old [17]; and (7) to ensure developmental homogeneity, only participants born at a gestational age ≥ 38 weeks were included, thereby excluding premature infants whose auditory pathway maturation may differ significantly from that of term neonates.

### 2.3. Procedures

All newborns and infants included as subjects in the study were evaluated through acoustic immittance measures (tympanometry and ipsilateral acoustic reflexes) and the transient otoacoustic emissions test, with responses within the normal limits in these tests. Individuals who presented any type of alteration at this stage were referred for otorhinolaryngological evaluation and management and were not included in the present study.

The acoustic immittance measurements were performed using the GSI TympStar Pro 2™ middle ear analyzer (Grason-Stadler, Eden Prairie, MN, USA). For infants under 6 months of age, a 1000 Hz probe was used, in accordance with literature recommendations [20], which indicate that high-frequency tympanometry has greater sensitivity in identifying middle ear changes in this age group due to the resonance characteristics of the external auditory canal and the middle ear, which differ substantially from those observed in older children and adults [21,22,23,24] (Alaerts et al., 2007; Baldwin, 2006; Holte et al., 1991, Kei et al., 2003). For individuals aged 6 months or older, the conventional 226 Hz probe was used, which is widely adopted in audiological clinical practice for this population [4,25,26]. For the 226 Hz probe, the tympanometry curve should have a compliance peak of 0.2–1.6 mmhos over the range of −100 to +200 daPa, which is the recommended value for children aged 6 months to 6 years [27]. Regarding this, the 1000 Hz probe’s tympanometric curve should present a single or double-peak trace within the pressure range of −200 to +200 daPa with a compliance peak greater than 0.2 mmhos [21,22,24].

The evaluation of transient otoacoustic emissions (TOAEs) was conducted after confirming the integrity of the tympanic-ossicular system with the NeuroAudio system (NeuroAudioTM—Neurosoft, Ivanovo, Russia) in an electrically shielded and acoustically attenuated environment. Click-evoked otoacoustic emissions were recorded using a nonlinear acquisition protocol. The stimulus was delivered at an intensity of approximately 80–84 dB peSPL across a 20 ms time window [16]. For each ear, data collection was completed upon the accumulation of 260 artifact-free sweeps, with the artifact rejection threshold configured at 47 dB SPL to prevent noise-contaminated epochs from being incorporated into the average, which is appropriate for neonates and young infants [28]. Prior to initiating each recording session, adequate probe fit was confirmed to guarantee stimulus delivery integrity. Response validity was determined by two criteria applied concurrently: a minimum signal-to-noise ratio (SNR) of 6 dB SPL, reflecting the pass threshold endorsed by the British Society of Audiology [27] (BSA, 2023) and a waveform reproducibility coefficient of at least 90%, a more stringent threshold adopted to strengthen the reliability of response detection within the scope of this investigation.

The ABR data collection took place in an electrically shielded and acoustically attenuated environment while the participant rested comfortably in a reclined position during natural sleep. Sedation was not required or administered for any participant in this study. The NeuroAudio system (NeuroAudio^TM^—Neurosoft, Ivanovo, Russia) was employed for ABR measurement. The equipment was calibrated according to the Neurosoft Neuro-Audio Calibration Guidelines (CG032.01.003.000), which follow the relevant international standards, including ISO 8253-1:2010 [29], IEC 60645-1:2017 [30], IEC 60645-3:2007 [31], and the ISO 389-2:1994 [29]. The ABR was recorded with a three-electrode setup. The non-inverting electrode was placed at the high forehead (Fz), the inverting electrode on the ipsilateral mastoid, and the ground electrode on the forehead [32]. Electrode impedance was <5 kΩ, and the inter-electrode impedance was maintained below 3 kΩ. Since all participants were evaluated during natural sleep, eye movement artifacts were inherently minimized. Recordings were initiated only after confirmed sleep onset, and any epochs contaminated by movement artifacts were automatically rejected by the system’s built-in artifact rejection algorithm. Stimuli consisted of a 0.1 ms, rarefaction polarity clicks presented monaurally via ER-3A insert earphones (Etymotic Research, Inc., Schaumburg, IL, USA). The presentation level was 80 dB nHL at a repetition rate of 19.3/s. The ear testing order was randomized across participants, with the right ear recorded first in 50% of the cases and the left ear recorded first in the remaining 50%. Two separate collections of 2000 artifact-free stimuli were averaged per ear. The recorded responses were band-pass filtered between 100 Hz and 3000 Hz.

The ABR waveforms were independently reviewed by two evaluators with training in auditory electrophysiology. In cases of visual ambiguity or discrepant peak identification between the two reviewers, a consensus decision was reached in consultation with a senior audiologist with more than 25 years of clinical experience in ABR interpretation, whose determination served as the reference standard. This structured dual-review protocol was applied consistently across all recordings to ensure reliable and consistent wave identification. The ABR waveforms were visually inspected to identify the primary, reliable wave peaks (I, III, and V) based on their characteristic latency windows and morphological features. Once the peaks were definitively identified, the following measures were systematically derived: the absolute latencies of waves I, III, and V; the interpeak intervals (I–III, III–V, and I–V); the interaural latency differences for wave V; and the amplitude ratio of waves V and I.

### 2.4. Statistical Analysis

Statistical analysis was performed to describe the data and to establish normative values for quantitative variables. One-way analysis of variance (ANOVA) was used to evaluate age-related differences and to verify the appropriateness of the initial age group division. The initial three-month age bins were defined a priori for recruitment purposes. The final consolidation into three age groups (0–3, 4–12, and 13–36 months) was determined analytically, according to two pre-specified criteria: (i) the pattern of statistically significant versus non-significant pairwise differences in wave V latency between adjacent bins (Games–Howell post hoc), and (ii) the requirement of a minimum sample size per group sufficient to derive stable normative limits. Adjacent bins that did not differ significantly from one another were merged, yielding the three maturationally and statistically coherent groups used in all subsequent analyses. When the assumption of homogeneity of variance was violated, Welch’s ANOVA was applied, and Games–Howell post hoc tests were used.

For inferential analyses, linear mixed-effects models (LMMs) were used to account for the non-independence of measurements obtained from both ears of the same child. ABR variables were entered as dependent outcomes. Ear (right and left) and sex were included as fixed factors, and age (in months) was included as a continuous covariate. Interactions between ear and sex, as well as between ear and age, were tested to investigate potential differential effects between groups. The participant identification number was included as a random intercept to model the within-subject correlation arising from repeated measurements obtained from both ears of the same participant. After the identification of statistically significant effects, post hoc multiple comparisons were conducted using the Bonferroni adjustment to account for Type I error. Each child contributed measurements from both ears. The mixed-model approach explicitly accounted for the paired structure of the data and the resulting within-subject correlation between ears. Models were estimated using restricted maximum likelihood (REML).

Model assumptions were evaluated through graphical inspection of residuals (Q–Q plots), normality tests (Shapiro–Wilk and Kolmogorov–Smirnov), and homogeneity of variances (Levene’s test). Due to the sample size, normality was primarily interpreted based on visual inspection of the residuals. Random-effects structures were evaluated through variance component estimates and intraclass correlation coefficients (ICCs). In cases of minor deviations from model assumptions, analyses were retained considering the documented robustness of linear mixed-effects models.

Additionally, analyses were conducted with age categorized into age groups (0–3, 4–12, and 13–36 months) to facilitate clinical interpretation of the results. Analyses were performed using jamovi (version 2.6.44), adopting a significance level of 5%. Graphical analyses were conducted using Excel (Microsoft Corporation, Redmond, WA, USA), version 365, and Microsoft Power BI (Microsoft Corporation, Redmond, WA, USA), version 2.155.756.0. Control for multiple comparisons was addressed by using the Games–Howell procedure for pairwise age-group contrasts, which corrects for both multiplicity and unequal variances. In addition, all effects reported as statistically significant in the linear mixed-effects models had *p*-values below 0.001, remaining well below a conservative Bonferroni-adjusted threshold for the family of ABR outcomes. This approach ensured that the main findings were not driven by an inflated Type I error.

## 3. Results

### 3.1. Selection and Effects of Age Groups

A total of 203 children (406 ears) younger than 3 years of age were included in the study. The group consisted of 121 males and 82 females. The age ranged from 2 weeks to 36 months (Mean = 16.0 months, SD = 11.1). Initially, the children were divided into 12 age groups with three-month intervals (from 2 weeks up to 36 months of age). Wave V latency was selected as the criterion variable for age group verification because it is the most robust and consistently identifiable component of the click ABR across all pediatric age groups, including neonates [12,15]. Wave I latency shows negligible age-related variation in the pediatric population and was therefore uninformative for group discrimination. Wave III, while age-sensitive, is occasionally absent or morphologically ambiguous in the youngest infants, which would reduce the number of usable observations for group-level analysis. The use of wave V latency as the primary stratification criterion is consistent with prior pediatric ABR normative studies [33,34]. To verify whether this detailed division was justified, an ANOVA was performed. The analysis showed a statistically significant effect of age on wave V latency (*F* (11,183) = 17.45; *p* < 0.001). As the assumption of homogeneity of variance was violated (Levene’s *p* = 0.009), Welch’s correction was applied. Welch correction confirmed the significant effect of age, *F* (11,64.95) = 16.12, *p* < 0.001. The effect size was large (partial *η*^2^ = 0.51). Post hoc comparisons (Games–Howell procedure) indicated that infants aged 0–3 months differed significantly from most of the older age groups (*p* < 0.001). No significant differences were found between the 4–6, 6–9, and 9–12-month groups. Age groups between 13 and 36 months showed no consistent significant differences. Considering both the statistical results and the relatively small number of children in some subgroups, age categories that did not differ significantly were combined. Finally, three age groups were established:

0–3 months (*n* = 36),

4–12 months (*n* = 53),

13–36 months (*n* = 114),

This classification reflects the maturational pattern of ABR and ensures sufficient sample size for establishing normative values. The final group sizes ranged from 36 to 114 participants. Linear mixed-effects models were conducted to investigate the effects of sex, ear, and age (in months) on the absolute latencies and interpeak intervals of the ABR, including the interactions between these factors. Table 1 presents the results of these models, in which the latencies of waves I, III, and V were analyzed, as well as the interpeak intervals I–III, III–V, and I–V, considering sex, ear, age (in months), and their interactions as independent variables.

**Figure 1:**
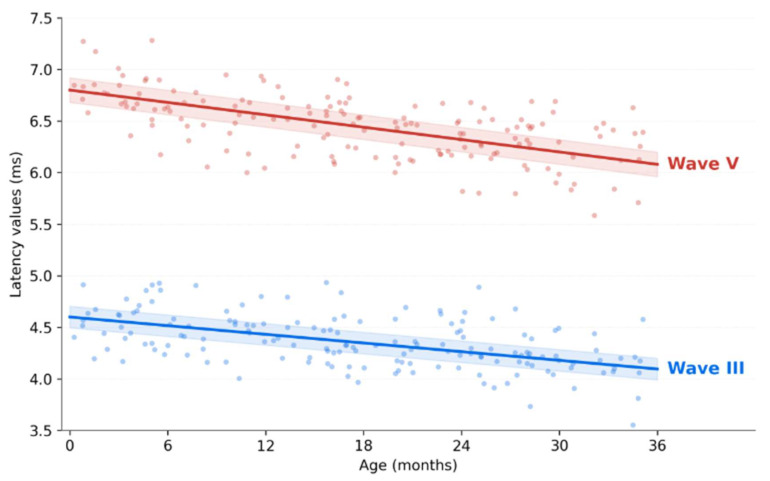
Developmental trajectory of waves III and V latencies across the first 36 months of life.

The analysis of age, considered as a continuous variable in months, showed a significant effect on the latencies of waves III and V, as well as on the interpeak intervals I–III, III–V, and I–V. It was observed that, for the latency of wave III, there is an average reduction of 0.014 ms for each month of life. Similarly, the latency of wave V showed an average reduction of 0.020 ms per month. Regarding the interpeak intervals, a decrease of 0.013 ms per month was observed for the I–III interval, 0.009 ms for the III–V interval, and 0.019 ms for the I–V interval. These findings are visualized in Figure 1, which illustrates the developmental trajectory of waves III and V latencies across the first 36 months of life, demonstrating the progressive reduction in latencies with advancing age.

The analysis by age groups also showed a significant effect of age on all ABR variables, except for the latency of wave I. For the latency of wave V, a significant effect of the age factor was observed (F = 74.44; *p* < 0.001), indicating a progressive reduction in latencies with increasing age. Compared to the group of 0 to 3 months, all other groups showed significantly lower values: 4 to 12 months (β = −0.36; Pbonferroni < 0.001) and 13 to 36 months (β = −0.69; pbonferroni < 0.001). Similar results were observed for the latency of wave III (F = 68.14; *p* < 0.001), with a significant reduction in the 4-to-12-month (β = −0.16; Pbonferroni < 0.001) and 13-to-36-month (β = −0.43; Pbonferroni < 0.001) groups compared to the younger group.

**Figure 2:**
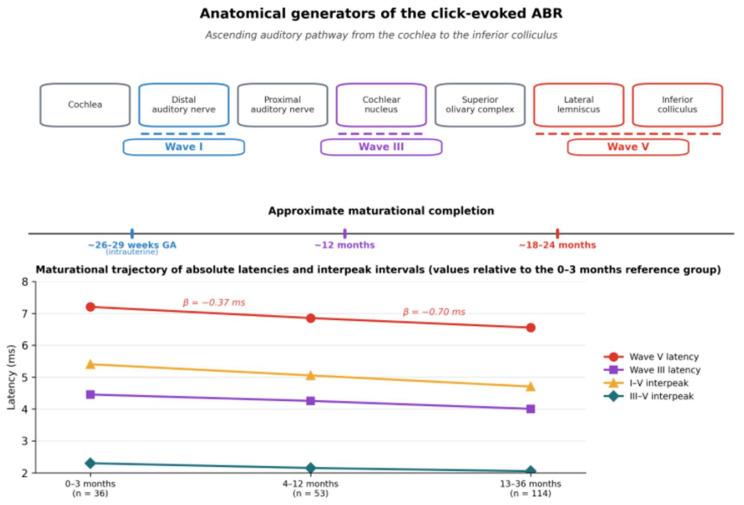
Anatomical generators of the click-evoked ABR along the ascending auditory pathway (cochlea to inferior colliculus) with approximate maturational completion timeline, and maturational trajectory of wave III and wave V latencies and of the I–V and III–V interpeak intervals across the three age groups defined in the present study. GA = gestational age.

Regarding the interpeak intervals, the I–III interval showed a significant age effect (F = 93.28; *p* < 0.001), with a progressive reduction in the 4-to-12 month (β = −0.14; Pbonferroni < 0.001) and 13-to-36-month (β = −0.39; Pbonferroni < 0.001) groups. The interpeak III–V also demonstrated a significant effect of age (F = 84.78; *p* < 0.001), with lower values in the groups of 4 to 12 months (β = −0.18; Pbonferroni < 0.001) and 13 to 36 months (β = −0.33; Pbonferroni < 0.001) when compared to the 0-to-3-month group. Similarly, the interpeak I–V showed a significant age effect (F = 90.34; *p* < 0.001), with a significant reduction in the 4-to-12-month (β = −0.34; Pbonferroni < 0.001) and 13-to-36 month (β = −0.64; Pbonferroni < 0.001) groups. The anatomical basis and maturational significance of these findings are illustrated in Figure 2, which presents the anatomical generators of the click-evoked ABR along the ascending auditory pathway, together with the maturational trajectories of waves III and V latencies and the interpeak intervals (I–V and III–V) across the three age groups.

### 3.2. Effect of Ear

Regarding the ear, no significant differences were identified for the latencies of waves I, III, and V, nor for the interpeaks I–III and I–V (*p* > 0.05). However, a significant effect was observed for the III–V interpeak (F = 811.68; *p* < 0.001), indicating that the right ear showed a latency value 0.46 ms lower than the left ear. The interaural difference for wave V did not show any statistically significant effect of age, sex, or their interactions. In children with normal hearing, wave V latency can therefore be considered bilaterally symmetric within the measurement variability of the NeuroAudio system, and interaural latency differences for this component were not reported in the normative tables.

### 3.3. Effect of Sex

Sex showed a consistent significant effect, influencing the latencies of waves III (F = 18.71; *p* < 0.001) and V (F = 27.39; *p* < 0.001) and all interpeak intervals (I–III: F = 28.47; *p* < 0.001; III-V: F = 29.49; *p* < 0.001; and I–V: F = 36.83; *p* < 0.001). The differences found indicated shorter latencies for female individuals. For the latency of wave I, no significant effect was observed (*p* = 0.756).

### 3.4. Effect of the Interaction Between Sex and Ear and Between Ear and Age

No statistical differences were observed for the interaction of sex and ear for any of the latencies or interpeak intervals (*p* > 0.05). Regarding the interaction between ear and age, a significant interaction was observed for the III-V interpeak interval(F = 32.39; *p* < 0.001), indicating that the difference between the ears varies depending on age.

### 3.5. Amplitude

The amplitude of wave I showed an average of 0.349 μV (SD = 0.165) in the right ear and 0.345 μV (SD = 0.164) in the left ear. For wave V, average values of 0.454 μV (SD = 0.171) were observed in the right ear and 0.450 μV (SD = 0.169) in the left ear. Overall, similarity between the ears was observed for both waves.

**Figure 3:**
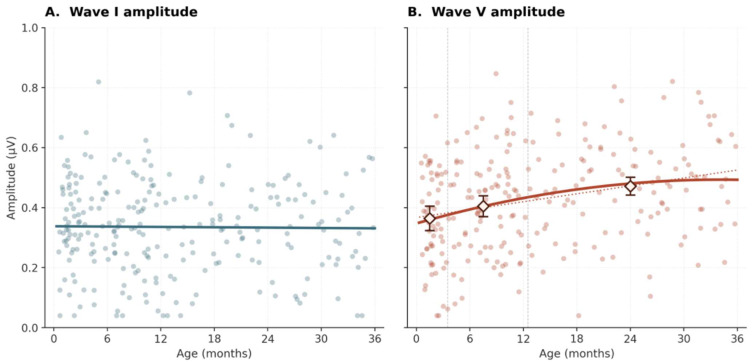
Comparison of the amplitude values of waves I and V considering the age group.

Linear mixed-effects models were conducted to investigate the effects of sex, ear, and age (in months) on the amplitude of the waves I and V of the ABR, including the interactions between these factors. For the amplitude of wave I, no statistically significant effects of sex, ear, or age were observed, nor any interactions between these variables (*p* > 0.05). Regarding the amplitude of wave V, a significant effect of age was observed (F = 12.81; *p* < 0.001), indicating a progressive increase in amplitude with advancing age. Specifically, an average increase of 0.005 μV per month of life was estimated. The age-related changes in wave amplitude are depicted in Figure 3, which shows the comparison of amplitude values for waves I and V across the three age groups, demonstrating the progressive increase in wave V amplitude with advancing age.

The analysis considering the categorized age (0–3, 4–12, and 13–36 months) corroborated the findings of the continuous analysis. Compared to the 0-to-3-month group, the 13-to-36-month group showed a significantly greater amplitude (β = −0.17; Pbonferroni = 0.003), corresponding to an approximate increase of 0.17 μV. Similarly, a significant difference was observed between the 4-to-12-month and 13-to-36-month groups (β = −0.10; Pbonferroni = 0.042), with a greater amplitude in the older group.

No statistically significant differences were identified between the 0-to-3-months and 4-to-12-months groups (*p* > 0.05). Together, these results indicate that the amplitude of wave V increases with age, with more evident differences between the first year of life and the older age groups.

### 3.6. Normative Values

Normative reference limits were computed from the normative sample as mean + 2 standard deviations and mean + 3 standard deviations for each age group and sex. The corresponding means and standard deviations are presented in Table 2 to allow independent verification of the reported cutoffs. Table 3 shows the separate 2 SD and Table 4 shows the 3 SD reference limits for click-evoked ABR latencies and interpeak intervals by age group and sex. 

## 4. Discussion

This study presents the first normative dataset for click ABR recorded with the NeuroAudio system (Neurosoft, Ivanovo, Russia) in children under three years of age, consisting of 203 participants categorized into three clinically and statistically validated age groups: 0–3, 4–12, 13–36 months. This contribution addresses a significant deficiency in the pediatric audiological literature, where equipment-specific normative references for this system are presently restricted to adult populations [35,36].

### 4.1. Selection and Effects of Age Groups

The categorization of age into groups was adopted with the aim of representing distinct stages of auditory development throughout the early years of life. Moreover, the use of age groups is particularly relevant for the construction of normative values, as latency and interpeak measures show significant variation with age. Thus, age stratification enables a more precise clinical interpretation appropriate to the individual’s stage of development, contributing to greater diagnostic accuracy.

It is important to mention that the integrity of the tympanic-ossicular system and cochlear function was determined prior to the evaluation of the ABR click in all individuals of the present study, as any type of alteration in these structures could compromise the quality of the ABR click responses. Alterations in the tympano-ossicular system due to conditions of ossicular chain fixation or even otitis media could directly impact click ABRs [37,38,39]. Simultaneously, a compromise in cochlear functionality, more specifically in the outer hair cells, which are pre-neural structures, could also influence the click ABRs.

The present study aimed to establish a guideline composed of neonates and children subdivided into 12 age groups with intervals of 3 months between the groups; however, statistical analyses revealed the need to reformulate the age groups. For this reason, there was a reformulation of the age groups, which are now: 0–3 months, 4–12 months, 13–36 months. The new age classification made it possible to observe a component of a maturational pattern, in which there is a reduction in the absolute latency values of waves III and V in the first months of life (0–3 months), with the absolute latency values of wave V showing a more pronounced reduction in this first trimester of life. These findings are in accordance with the literature, since the structure involved in the generation of wave I is the auditory nerve, which undergoes a complete myelination process around the 26th to 29th weeks of gestation, that is, still in intrauterine life [40]. The low variability of wave I across age groups can be explained by the maturation pattern of the auditory nerve, which, in addition to earlier maturation, remains more stable than the other waves of the click ABR throughout life [33,41]. From this perspective, a change in the absolute latency values of wave I, in the presence of integrity in the tympanic-ossicular system and cochlear functionality, could be a strong indicator of significant impairment in auditory information processing and should serve as a warning within the diagnostic process [36].

A consistent pattern of reduced latencies and interpeaks with increasing age was observed, with greater differences concentrated between the 0-to-3-months group compared to the other age groups. The results demonstrate a clear pattern of auditory neurological maturation in the first 36 months of life, reflected by the progressive reduction in absolute latencies of waves III and V and the interpeak intervals I–III, III–V, and I–V with advancing age. This pattern is consistent with the process of myelination of the auditory pathways of the brainstem, which begins in the prenatal period and extends throughout the early years of life. The absence of a significant effect of age on the latency of wave I reinforces that peripheral structures (auditory nerve/cochlea) already reach relative maturity early, while central pathways, reflected by waves III and V, continue to mature actively [40,42].

### 4.2. Effect of Ear

The results of the ear analysis demonstrated that there is a correlation between the performance of the right and left ears. Eldredge and Salamy [43] reported the existence of differences in the interpeak interval values between the ears, with earlier values in the right ear even in neonates, corroborating our findings. For researchers, the III-V interpeak interval is the most sensitive to the immaturity of the brainstem. This means that the III-V is in the period of greatest maturational variability in this age group (<3 years), making it more susceptible to revealing asymmetries.

Currently, the interaural interaction difference is frequently used considering the latency values of wave V [12,44,45]. Our results showed that the analysis of differences between the ears can be performed by considering the differences in the latency values of waves I, III, and V, as well as the values of the interpeak intervals I–III and I–V. They also emphasize that interaural difference values should not be considered in the analysis of III–V interpeak response differences, since, in neonates and infants with normal hearing, there can be an average difference of 0.46 ms, with better performance of the right ear.

There is a gap in the literature regarding the normative values of wave III in both the pediatric population and in adults and the elderly. In the studies by Sanfins et al. [46], interaural differences in wave III latencies were reported to be significantly affected by auditory asymmetry, but not by sex or age [47]. On the other hand, Sanfins et al., shows that interaural asymmetry in interpeaks involving wave III has functional significance. The present study presents scientific relevance with the normative analysis of latency and amplitude values of wave III, interpeak interval I-III and III-V in a robust and stratified pediatric sample, and the data support that wave III and its interpeaks (I–III, III–V) are sensitive to peripheral asymmetry and that interaural differences in wave III are not merely reflective but can indicate some type of dysfunction in the integrity of the auditory pathway. However, it is recommended that new studies with this population be conducted to verify these results.

Stapells et al. (1982) [45] demonstrated that the interaural differences for wave V in children from three months to three years are similar to those of newborns and adults, suggesting that auditory symmetry is established early and is maintained throughout development. Thus, the results of the present study are in accordance with the global literature regarding the importance of wave V latency values between the right and left ears.

The existence of an interaural difference in the III–V interpeak interval with lower latency values for the right ear has been discussed in the literature in relation to the medial olivocochlear (MOC) system. Although not directly assessed in the present study, one plausible mechanistic framework is that the MOC asymmetrically activates both ears, such that the left ear experiences greater contralateral suppression than the right ear [48,49]. This implies that, in the efferent pathway, the left ear receives more olivocochlear inhibition, which may result in differentiated pre-neural modulation that is indirectly reflected in the processing time of the III–V interpeak interval. If the right cochlear input reaches the cochlear nucleus with a slight temporal or neural synchronization advantage, even if not sufficient to differentiate waves I or III individually, this small difference may be amplified along the lateral lemniscus synapses to the inferior colliculus, resulting in a shorter III–V interval on the right side [50,51,52]. It should be emphasized, however, that this interpretation remains hypothetical in the absence of direct contralateral suppression OAE measurements and warrants prospective investigation. A difference in responses in the III–V interval may therefore be related to the development of auditory skills that, over the years, would achieve equity between the ears.

### 4.3. Effect of Sex

The current study reveals that sex appears to have an effect on click ABRs, with wave I being the sole component that does not appear to be affected by sex. Sininger et al. [53], highlight similar differences in both adults and newborns, which is consistent with their findings. The disparities in responses between the sexes are widely documented in research utilizing click ABRs. These discrepancies can be explained by physical difficulties such as the length of the cochlea, which is approximately 13% shorter in females compared to males. This is also associated with the smaller head size typically found in females [54].

At the same time, it has been observed that there are no differences in the latency values of wave I across individuals of varying ages and across studies utilizing a variety of approaches.

The current study provides robust evidence supporting the stability of wave I latency across early development. This finding is consistent with the established literature demonstrating that wave I responses reach functional maturity by birth due to the prenatal completion of auditory nerve myelination [55,56,57], emphasizing the reliability of wave I responses from the neonatal period onward. As a result, deviations in wave I latency in the presence of normal middle ear function may serve as an early indicator of auditory nerve or cochlear dysfunction [15,58]. The observed reduction of approximately 0.014 ms per month in wave III latency and 0.020 ms per month in wave V latency aligns closely with maturational patterns documented in prior normative studies [15,59,60], reflecting the progressive myelination and synaptic refinement of central auditory pathways during the first three years of life.

Based on these findings, it appears that the delay values of waves III and V make it possible to monitor the process of neuromaturation, which is of utmost significance in neonates and newborns. Because of this, these parameters can be considered a biomarker of auditory neuromaturation. This allows for the evaluation and monitoring of the progressive myelination of the auditory pathways, as well as the neural refinement of the structures that are located in the brainstem region [36,61].

Due to the fact that the development of peripheral and central auditory pathways essential for the processing of speech and language, learning, and social connections of a child, this monitoring is of utmost importance [62,63]. It is essential to continue the investigation process in order to ensure the best conditions for auditory, cognitive, and language development. In this context, the evaluation of the click ABR can serve as a preliminary tool to assess auditory maturation. If there is any deviation from the normative values for each age group, it is essential to continue the investigation process.

### 4.4. Effect of the Interaction Between Sex and Ear and Between Ear and Age

The age range investigated in this study coincides with the critical period of maturation of the brainstem auditory pathway. The III–V interpeak interval reflects neural conduction between the cochlear nucleus (the generator of wave III), located in the caudal portion of the pons, and the lateral lemniscus and inferior colliculus, which correspond to the generation of wave V. Therefore, it is a higher area of the auditory system that progresses and matures more slowly and progressively throughout the first year of life [40,64].

Moreover, the significant interaction observed between ear and age in the III–V interpeak is consistent with the hypothesis that auditory subcortical asymmetry is not a static phenomenon but a dynamic process that modulates throughout development and may represent a precursor to hemispheric specialization for the processing of auditory and speech information [65]. Although not directly tested in the present study, this interpretation is supported by converging evidence in the literature: Ruthig et al. (2025) [66] demonstrated structural hemispheric asymmetry in intracortical myelin orientation within the mouse auditory cortex, suggesting that lateralized myelination patterns may have a neuroanatomical basis. This interpretation, however, remains hypothetical in the human pediatric context and warrants prospective investigation combining ABR and neuroimaging measures. The meta-analysis by Stipdonk et al. (2016) [34] reinforces that the III–V interpeak is the parameter that most discriminates atypical maturational trajectories, with a large effect size compared to other ABR components.

Thus, the interaction observed in the present study does not represent an isolated finding but is part of a body of evidence that supports that (i) myelination and synchronization of the central auditory pathways in the brainstem occur in a lateralized manner [65,66]; (ii) this lateralization is more evident in the rostral segments (III–V) than in the caudal ones (I–III) [65,67]; and (iii) the temporal dependence of this asymmetry, detected here as ear × age interaction, may reflect the very dynamic process of establishing auditory hemispheric dominance over the first three years of life [68,69].

### 4.5. Amplitude

The analysis of amplitude values demonstrated that wave I remained stable in relation to age, sex, and ear, whereas wave V showed a progressive increase with advancing age; therefore, there is a differentiated pattern between the waves. This pattern of responses also seems to be consistent with the maturational aspect, since the generators of wave I are located more peripherally in relation to wave V, just as observed for latency [70]. Moreover, the stability of wave I amplitude values also supports that the normative values of this component should be considered a biomarker of neuromaturation in neonates and infants.

Another highlighted aspect was that the amplitude of wave V occurred exclusively in the group aged 13 to 36 months compared to the younger age groups, suggesting that the main maturational increase in amplitude occurs during the second and third years of life, due to a slow trajectory resulting from the gradual increase in the number of synchronized fibers that contribute to the auditory evoked potential response [71]. Our study reports an increase of 0.005 µV per month, equivalent to 0.18 µV over 36 months of age, representing an increase in neuronal activity with age in the amplitude of wave V.

The absence of a significant interaction between age and sex, or between age and ear for the amplitude of wave V indicates that the maturational trajectory of the amplitude is a robust and uniform phenomenon across sexes and ears. Therefore, this reinforces the idea that babies need to be stimulated so that sound signals traverse the auditory system and greater neuronal stimulation occurs.

### 4.6. Normative Values

There is a consensus in the specialized literature that the interpretation of the ABR click should consider different normality criteria based on the age range. For individuals above 2 or 3 years old, the use of two standard deviations (SD) is considered a robust metric [12,34,36,72], On the other hand, in the pediatric population, the adoption of three standard deviations is recommended, aiming to accommodate the greater intersubject variability typical of this phase. The use of a wider confidence interval (3 SD) in infants increases clinical accuracy in differentiating physiological maturational variations from possible pathological changes, especially in cases of prematurity [10,12,73].

### 4.7. Study Limitations

This study employed a convenience sampling strategy at a single academic audiology center, which may introduce selection bias. The cohort was limited to families with access to tertiary audiology services and comprised only typically developing children with confirmed normal peripheral hearing and middle ear function. These characteristics may restrict the representativeness of the normative data regarding socioeconomically and geographically diverse populations. Future multi-center studies with broader recruitment strategies would strengthen the generalizability of these reference values. It is worth noting that this structural characteristic is not unique to the present study: the Boys Town normative dataset [14,15], which remains the most widely cited pediatric ABR reference worldwide, was similarly derived from a single institutional cohort. Additionally, the 0–3-month age group presented a smaller sample size (n = 36) relative to the older cohorts, a limitation inherent to the recruitment constraints of this age range, including the requirement of natural sleep, parental scheduling, and strict audiological eligibility criteria. Although the large effect size for age (partial η^2^ = 0.51) supports the statistical adequacy of the current sample, expansion of this youngest cohort in future studies would further consolidate the normative reference values for the most clinically critical developmental window. Future normative studies should incorporate explicit inter-rater reliability metrics to quantify the reproducibility of peak identification. Furthermore, the age group structure was derived using wave V latency as the primary stratification criterion. Although the resulting three-group solution was subsequently validated by consistent and significant age effects across all age-sensitive ABR variables, including wave III and V latencies, the interpeak intervals I–III, III–V, and I–V, and wave V amplitude, a prospective multivariate clustering approach applied simultaneously to all ABR variables would represent a more data-driven methodological alternative and is recommended for future normative studies in this field.

### 4.8. Final Comments

In general, the findings indicate that in this sample, age constituted the main factor associated with variations in the ABR measures, reflecting the auditory maturation process, followed by the effect of sex, while the influence of the ear was found to be restricted to the III–V interpeak, including an interaction with age in this same measure. Thus, the normative values of the click ABR in the pediatric population should be stratified by age and by ear for the III–V interpeak.

The age range 0–3 months represents the period of greatest immaturity in auditory life with the highest latency values of waves III and V. From the perspective of possible markers of this auditory maturation, the empirical data suggest that the I–V interval reflects the development and functioning of the structures involved in the generation of the ABR click, given that age was a very strong determinant in the interpeak I–V interval response during the first 36 months of life.

The age cohort of 13–36 months exhibited comparable values across all analyzed variables, indicating that the majority of auditory pathway maturation up to the brainstem, as assessed by click ABR, transpires prior to 13 months, with only minor alterations thereafter, particularly in amplitude values, which appear to be the most significantly affected beyond this age. Therefore, any changes in this age group should not be regarded as a deficiency of the auditory nerve system but should be examined with the highest diligence.

## 5. Conclusions

This study established normative reference data for click ABR recorded with the NeuroAudio system in children under three years of age, using a selected sample with normal peripheral hearing and tympanometry/otoacoustic emission findings. By confirming the expected maturational pattern, stable wave I latencies alongside progressive shortening of waves III and V and all interpeak intervals across increasing age, the results support the stratification of children into clinically meaningful age groups (0–3, 4–12, and 13–36 months), aligned with auditory brainstem maturation. The normative values proposed here (2 SD and 3 SD limits) provide a critical, equipment-specific framework for interpreting pediatric ABR recordings obtained with the NeuroAudio system, reducing the risk of misclassification associated with the use of adult or non-system-specific reference values. These findings strengthen the accuracy of early diagnosis and monitoring of auditory pathway integrity in infants and young children, and support the inclusion of sex- and ear-specific normative values for the III–V interpeak interval as standard practice in pediatric electrophysiological evaluation.

## Figures and Tables

**Table 1:** Results of the linear mixed-effects models for the latencies of waves I, III, and V and the interpeak intervals I–III, III–V, and I–V, considering the effects of sex, ear, and age (in months), as well as the interactions sex × ear and ear × age.

Variable	ICC	Significant Effect	β	95% CI	*p*-Value
Wave I	0.714	Age	−9.98 × 10^−4^	−0.002 to 7.28 × 10^-4^	0.257
		Sex (F—M)	−0.006	−0.045 to 0.032	0.756
		Ear (RE—LE)	0.028	−0.001 to 0.057	0.058
		Age × Ear (RE—LE)	−2.43 × 10^−4^	−0.001 to 0.001	0.739
		Sex (F—M) × Ear (RE—LE)	0.030	−0.002 to 0.062	0.069
Wave III	0.840	Age	−0.014	−0.016 to −0.011	**<0.001**
		Sex (F—M)	−0.140	−0.204 to −0.076	**<0.001**
		Ear (RE—LE)	0.015	−0.003 to 0.034	0.110
		Age × Ear (RE—LE)	3.84 × 10^−4^	−0.001 to 0.002	0.656
		Sex (F—M) × Ear (RE—LE)	0.012	−0.026 to 0.050	0.531
Wave V	0.863	Age	−0.020	−0.024 to −0.016	**<0.001**
		Sex (F—M)	−0.256	−0.352 to −0.159	**0.002**
		Ear (RE—LE)	−0.001	−0.027 to 0.024	0.918
		Age × Ear (RE—LE)	0.002	−2.95 × 10^−4^ to 0.004	0.087
		Sex (F—M) × Ear (RE—LE)	0.025	−0.027 to 0.079	0.340
Interpeak I–III	0.759	Age	−0.013	−0.015 to −0.010	**<0.001**
		Sex (F—M)	−0.134	−0.183 to −0.084	**<0.001**
		Ear (RE—LE)	−0.015	−0.033 to 0.003	0.104
		Age × Ear (RE—LE)	6.90 × 10^−4^	−9.56 × 10^−4^ to 0.002	0.411
		Sex (F—M) × Ear (RE—LE)	−0.017	−0.054 to 0.020	0.369
Interpeak III–V	0.277	Age	−0.009	−0.011 to −0.007	**<0.001**
		Sex (F—M)	−0.119	−0.162 to −0.076	**<0.001**
		Ear (RE—LE)	−0.466	−0.498 to −0.434	**<0.001**
		Age × Ear (RE—LE)	0.008	0.005 to 0.011	**<0.001**
		Sex (F—M) × Ear (RE—LE)	0.001	−0.064 to 0.066	0.970
Interpeak I–V	0.848	Age	−0.019	−0.022 to −0.015	**<0.001**
		Sex (F—M)	−0.251	−0.332 to −0.169	**0.017**
		Ear (RE—LE)	−0.028	−0.090 to 0.027	0.296
		Age × Ear (RE—LE)	0.002	−0.003 to 0.007	0.444
		Sex (F—M) × Ear (RE—LE)	−0.007	−0.055 to 0.039	0.750

Legend: ICC = Intraclass Correlation Coefficient; β: regression coefficient; CI = confidence interval; RE = Right Ear; LE = Left ear; F = Female; M = Male; *p* < 0.05 considered significant (bold).

**Table 2:** Normative descriptive statistics (ms) for click-evoked ABR latencies and interpeak intervals by age group and sex.

Age Group	Sex	Mean Wave I	SD Wave I	Mean Wave I (amp)	SD Wave I (amp)	Mean Wave III	SD Wave III	Mean Wave V	SD Wave V	Mean Wave V (amp)	SD Wave V (amp)	Mean I–III	SD I–III	Mean III–V (RE)	SD III–V (RE)	Mean III–V (LE)	SD III–V (LE)	Mean I–V	SD I–V
0–3 months	M	1.48	0.158	0.281	0.119	4.20	0.183	6.49	0.350	0.315	0.110	2.72	0.174	2.27	0.238	2.32	0.299	5.01	0.311
	F	1.42	0.473	0.372	0.162	4.13	0.164	6.26	0.210	0.360	0.858	2.71	0.156	2.10	0.124	2.13	0.144	4.82	0.192
4–12 months	M	1.42	0.146	0.332	0.144	4.04	0.238	6.08	0.258	0.416	0.945	2.62	0.159	1.99	1.68	2.08	0.158	4.65	0.223
	F	1.42	0.184	0.345	0.185	3.94	0.248	5.92	0.338	0.409	0.116	2.51	0.190	1.97	0.103	1.98	0.155	4.49	0.276
13–36 months	M	1.40	0.113	0.368	0.177	3.78	0.185	5.77	0.294	0.491	0.185	2.38	0.145	1.99	0.192	1.98	0.209	4.36	0.241
	F	1.40	0.130	0.310	0.143	3.67	0.206	5.56	0.273	0.547	0.214	2.26	0.153	1.88	0.167	1.90	0.180	4.15	0.231

Legend: SD = standard deviation; RE = right ear; LE = left ear; lat = latency; amp = amplitude; M = male; F = female.

**Table 3:** Absolute 2 SD reference limits (ms) for click-evoked ABR latencies and interpeak intervals by age group and sex.

Age Group	Sex	Wave I Lat (2 SD Limit)	Wave III Lat (2 SD Limit)	Wave V Lat (2 SD Limit)	I–III (2 SD Limit)	III–V (RE, 2 SD Limit)	III–V (LE, 2 SD Limit)	I–V (2 SD Limit)
0–3 months	M	1.16–1.80	3.83–4.57	5.79–7.19	2.37–3.07	1.79–2.75	1.72–2.92	4.39–5.63
	F	1.33–1.51	3.80–4.46	5.84–6.68	2.40–3.02	1.85–2.35	1.84–2.42	4.44–5.20
4–12 months	M	1.13–1.71	3.56–4.52	5.56–6.60	2.30–2.94	1.65–2.33	1.76–2.40	4.20–5.10
	F	1.05–1.79	3.44–4.44	5.24–6.60	2.13–2.89	1.76–2.18	1.67–2.29	3.94–5.04
13–36 months	M	1.17–1.63	3.41–4.15	5.18–6.36	2.09–2.67	1.61–2.37	1.56–2.40	3.88–4.84
	F	1.14–1.66	3.26–4.08	5.01–6.11	1.95–2.57	1.55–2.21	1.54–2.26	3.69–4.61

Legend: 2 SD = 2 standard deviation; RE = right ear; LE = left ear; lat = latency; M = male; F = female.

**Table 4:** Absolute 3 SD reference limits (ms) for click-evoked ABR latencies and interpeak intervals by age group and sex.

Age Group	Sex	Wave I Lat (3 SD Limit)	Wave III Lat (3 SD Limit)	Wave V Lat (3 SD Limit)	I–III (3 SD Limit)	III–V (RE, 3 SD Limit)	III–V (LE, 3 SD Limit)	I–V (3 SD Limit)
0–3 months	M	1.01–1.95	3.65–4.75	5.44–7.54	2.20–3.24	1.56–2.98	1.42–3.22	4.08–5.94
	F	1.28–1.56	3.64–4.62	5.63–6.89	2.24–3.18	1.73–2.47	1.70–2.56	4.24–5.40
4–12 months	M	0.98–1.86	3.33–4.75	5.31–6.85	2.14–3.10	1.49–2.49	1.61–2.55	3.98–5.32
	F	0.87–1.97	3.20–4.68	4.91–6.93	1.94–3.08	1.66–2.28	1.52–2.45	3.66–5.32
13–36 months	M	1.06–1.74	3.23–4.34	4.89–6.65	1.95–2.82	1.41–2.57	1.35–2.61	3.64–5.08
	F	1.01–1.79	3.05–4.29	4.74–6.38	1.80–2.72	1.38–2.38	1.36–2.44	3.46–4.84

Legend: 3 SD = 3 standard deviation; RE = right ear; LE = left ear; lat = latency; M = male; F = female.

## Data Availability

The raw data supporting the conclusions of this article will be made available by the authors upon request due to privacy concerns.

## Abbreviations

The following abbreviations are used in this manuscript:

ABR	Auditory Brainstem Response
ANOVA	Analysis of variance
ASHA	American Speech-Language-Hearing Association
BSA	British Society of Audiology
daPa	Decapascal
dB	Decibels
CI	Confidence Interval
EPM	Paulista School of Medicine
ER—3A	Etymotic Research Insert Earphones Model 3A
F	Female
Fz	Frontal Zero Electrode Position
GSI	Grason-Stadler Inc.
Hz	Hertz
ICC	Intraclass Correlation Coefficient
ICF	Informed Consent Form
JCIH	Joint Committee on Infant Hearing
kΩ	Kilo-ohm
LE	Left Ear
LMM	Linear Mixed-effects Models
M	Male
Mmhos	Millimhos
MOC	Medial Olivocochlear
ms	Millisecond
nHL	Normalized Hearing Level
peSPL	Peak equivalent Sound Pressure Level
RE	Right Ear
REC	Research Ethics Committee
REML	Restricted Maximum Likelihood
SD	Standard Deviation
SF-ABR	Specific Frequency Auditory Brainstem Response
SNR	Signal-to-noise ratio
SPL	Sound Pressure Level
TOAE	Transient otoacoustic emissions
UNIFESP	Federal University of São Paulo
µV	Microvolt
η^2^p	Partial eta-squared
